# Profile of Skin Disorders in Unreached Hilly Areas of North India

**DOI:** 10.1155/2016/8608534

**Published:** 2016-09-21

**Authors:** Deepak Dimri, Venkatashiva Reddy B, Amit Kumar Singh

**Affiliations:** ^1^Department of Dermatology, Veer Chandra Singh Garwhali Government Medical Sciences and Research Institute, Uttarakhand, India; ^2^Department of Community Medicine, Veer Chandra Singh Garwhali Government Medical Sciences and Research Institute, Uttarakhand, India

## Abstract

*Background.* The pattern of skin morbidity in an area depends on climate, geography, socioeconomic status, nutrition, genetics, and habits of the community.* Objective.* The objective of the present study was to describe the morbidity profile of patients attending dermatology outpatient department in a tertiary care centre of Garhwal hills, North India.* Methodology.* This is a record based study carried out using the morbidity registers. Patient details, diagnosis, and treatment provided by physicians were documented in the morbidity register. ICD coding was done to categorize the patients.* Results.* The total number of new episodes of illnesses treated in the skin outpatient department during 2009–2014 was 47465. Adults (>18 years) constituted about 80.9%. Among adults, about 59.9% were males. Overall the infections of the skin and subcutaneous tissue were the most common (32.6%) followed by the disorders of skin appendages (19.8%), and dermatitis and eczema (18.8%). Of the total patients 16.9% were affected by dermatitis and 16.7% by acne. Psoriasis, urticaria, melasma, and vitiligo were present in 3.4%, 3.4%, 3.6%, and 3.3% patients, respectively.* Conclusion.* This knowledge will help in planning appropriate range services to meet the patients' needs and help in training of health staff to meet these needs.

## 1. Introduction

The pattern of skin morbidity in an area largely depends on its climate and geography. Burden of various skin diseases is determined by the socioeconomic status, nutrition, genetics, and habits of the community [1]. The prevalence of skin diseases in the general population varies from 6.3% to 11.2% [2]. Moreover, in developing countries, poor hygiene, lack of basic amenities, and overcrowding also play significant role in occurrence of few skin diseases [3, 4]. In addition, profile of skin disorder among patient is influenced by the distance needed to travel to seek health care in hilly terrains [5].

Uttarakhand also known as Uttaranchal is primarily a hilly state situated in northwest region of India. It is divided into two regions, namely, Kumaun and Garhwal. Uttarakhand comprises 13 districts (7 in Garhwal region and 6 in Kumaon). The districts in Garhwal region are, namely, Dehradun, Haridwar, Pauri, Tehri, Rudraprayag, Chamoli, and Uttarkashi. They constitute over 60 percent of the geographical area of the state, with over 80 percent covered by mountains. Veer Chandra Singh Government Medical Sciences and Research Institute, Srinagar, is one of the referral centres for Garhwal division. It is important to understand the terrain due to different ethnic and racial conditions and different culture of the population, which affect the pattern of the skin diseases [6].

An in-depth knowledge of the distribution and magnitude of skin problems is essential for understanding the burden of various diseases in the population. In addition, the knowledge of the burden of skin diseases will help in providing efficient health services to the community [7]. However, hardly any Indian studies were available from Garhwal region of Uttarakhand to understand epidemiology of skin diseases. The objective of the present study was to describe the morbidity profile of patients attending dermatology outpatient department in a tertiary care centre of Garhwal hills, North India.

## 2. Materials and Methods

This is a record based study carried out using the morbidity registers maintained for outpatients at Dermatology Department, Medical College Hospital, Veer Chandra Singh Garhwali Government Medical Sciences and Research Institute in Garhwal region, Uttarakhand, North India. Patient details, diagnosis, and treatment provided by physicians were documented in the morbidity register. Information like age, gender, residence, new or old case, and principal diagnosis was extracted from the registers using a data extraction sheet. The diagnosis was coded as per International Classification of Disease (ICD) 10 classification of 2010 by two doctors independently [8]. Newly registered outpatients between February 2009 and December 2014 were included in the study. Those outpatients who visited for follow-up of acute illnesses and chronic conditions were excluded. Descriptive analysis was done with SPSS version 17.0 (Chicago, IL, USA). Patients less than 18 years of age were considered as children, and proportions were given wherever necessary. All necessary permissions were obtained from the appropriate authority.

## 3. Results

Skin patients comprised 4.5% of all the cases who attended the hospital in study period. The total number of new episodes of illnesses that were treated in the skin outpatient department (OPD) during 2009–20114 was 47465. Adults (>18 years) constituted about 80.9%. Among children, males (49.3%) and females (50.7%) constituted nearly equal proportions, but among adults, about 59.9% were males. All disorders were broadly classified into eight ICD categories.

Overall the infections of the skin and subcutaneous tissue were the most common (32.6%) followed by the disorders of skin appendages (19.8%), dermatitis and eczema (18.8%), other disorders of skin and subcutaneous tissue (13.7%), and miscellaneous disorders (7.1%) (Table 1). Among the infections of the skin and subcutaneous tissue the most common was fungal infection (46.8%) followed by scabies and viral and bacterial skin diseases. Tenia dermatoses constituted 76.4% of all fungal infections. Of the total patients 16.9% were affected by dermatitis and 16.7% by acne. Psoriasis, urticaria, melasma, and vitiligo were present in 3.4%, 3.4%, 3.6%, and 3.3% patients, respectively (Table 2).

In adults (18–60 years), the most common diseases were the infections of the skin and subcutaneous tissue (in males 40.1%, in females 23.7%). The five most common diseases among adult males were fungal skin infections (21.0%), dermatitis (15.0%), acne (14.5%), other disorders of skin and subcutaneous tissue (9.6%), and scabies (7.4%). The corresponding diseases among adult females were acne (20.4%), dermatitis (18.8%), other disorders of skin and subcutaneous tissue (16.8%), fungal skin infections (12.0%), and viral skin diseases (4.7%) (Table 2). There was no significant difference of morbidity pattern between adult males and females (*p* = 0.48).

Among the paediatric cases, the most common diseases were infections of the skin and subcutaneous tissue (in boys 38.1%, in girls 29.5%) followed by disorders of skin appendages (in boys 19.3%, in girls 24.4%) and other disorders of skin and subcutaneous tissue (in boys 16.2%, in girls 18.8%). Among male children, the other common diseases were acne (17.6%) and fungal skin infections (12.4%). Among female children, the other common diseases were acne (21.4%) and dermatitis (12.5%). There was no significant difference of morbidity pattern between male and female child (*p* = 0.56).

Among the male elderly (more than 60 years of age) cases, the most common diseases were dermatitis and eczema (38.0%) followed by infection of the skin and subcutaneous tissue (35.5%). Among elderly female, the common diseases were infection of the skin and subcutaneous tissue (38.8%) and dermatitis and eczema (20.4%) (Table 1). No significant difference of morbidity pattern between elderly males and females was observed (*p* = 0.32).

Patients from different districts most commonly presented with primarily fungal infection of the skin and subcutaneous tissue, followed by dermatitis and eczema, disorders of skin appendages, mainly acne, and other disorders of skin and subcutaneous tissue, mostly melasma and vitiligo (Table 3). No significant difference of morbidity pattern among various regions was observed (*p* = 0.41).

## 4. Discussion

Our study outlines the spectrum of skin problems that presented to our tertiary care hospital during the years 2009–2014. The results of the present study showed that commonly diagnosed diseases were the infection of the skin and subcutaneous tissue, the disorders of skin appendages, dermatitis and eczema, and other disorders of skin and subcutaneous tissue. Among the infection of the skin and subcutaneous tissue the most common was fungal infection followed by scabies. Similarly, another study conducted in Kolkata found infective dermatoses (36.4%) to be the most common diagnosis [9]. A study from Gurgaon, India, also reported infective dermatoses to carry higher incidence than noninfective dermatoses [10]. On the contrary a study from Kumaun region of Uttarakhand found that 58.7% patients were having noninfective skin diseases while only 27.1% patients were suffering from infective skin diseases [5]. A study from northeast also found that the incidence of noninfectious dermatoses (58.07%) was slightly higher than that of infectious dermatoses [11].

Occurrence of fungal infection (15.3%) in present study was similar to another study conducted in Pune [12]. But it was higher compared to another study from an urban institute in Kolkata where 9.7% of cases were of fungal infections [13]. Comparably, tinea dermatophytosis was the commonest fungal infection followed by tinea versicolor in present study and hospital based study from Guwahati [14]. Among children, the skin and subcutaneous infections were the most common diagnoses. Two other similar studies from Indian subcontinent were carried out among children in Saurashtra and Pakistan and showed that skin infection comprised 83.3% and 60% of skin diseases, respectively [15, 16]. Higher proportion of the skin and subcutaneous infections among children is comparable, to a community based cross-sectional study done in Wardha district in Maharashtra, Central India, on 666 children aged 0–14 which found that infective dermatoses contributed 63.5% of all dermatoses [17]. Scabies was found in less than 10% of all new cases in the present study. Likely among all cases scabies was present in 8.9% in study from Imphal [4]. From a study from Mangalore, Karnataka, among the infective disorders, 9.4% of scabies cases were reported [18]. The high incidences of infections in our study may be due to the warm and highly humid climate and low socioeconomic status of such patients, more so belonging to hilly areas. The scarcity of clean water may play a critical role in this regard.

Unlike other studies dermatitis and eczema were the second most common diagnosis among skin patients. Eczemas was top diagnosis, comprising 23.1% and 19.9% of new cases in a study carried out in Guwahati [14] and Haldwani, Uttarakhand, respectively [5]. Although papulosquamous disease was seen in 4.5% of total cases, psoriasis was the commonest (3.4%). In the papulosquamous group the incidence of psoriasis was highest as stated by other hospital based study from Nepal [19]. The percentage of acne in present was found to be higher compared to a hospital based study where acne vulgaris contributed to 4.1% of cases; this difference could be due to difference in diagnostic technique. In the present study, we identified acne by looking at patients skin [20]. Analogous proportion of cases of vitiligo and urticaria was stated from the study done in Kerala, 4.7% and 3.5%, respectively [20]. The results of studies from Mali and Ethiopia are similar [21, 22].

There is paucity of population-based data on skin morbidity in general population of north India. The majority of related studies were health facility or school based, and not community based. The present study stated the expressed needs of the people instead of real burden of skin morbidity. Adequate population-based database on dermatoses in the general population is necessary to estimate the burden and required interventions.

The present study was conducted at tertiary care hospital in north India and hence it cannot be generalized to all of India. Though the morbidity profile was stratified by age, gender, and residence, it was not possible to classify on socioeconomic structure and seasonal variation. Age could not be ascertained in small proportion of patients. Although two physicians independently coded data, misclassification could have occurred during coding. However, the number of episodes of illnesses included in the study is large and is a strength of the study.

## Figures and Tables

**Table 1:** Pattern of skin diseases among patients at dermatology OPD, tertiary care hospital, Srinagar, Uttarakhand.

Serial number	ICD category	Disease	Value	Percentage
1	Infections of the skin and subcutaneous tissue (L00–L08)	Bacterial diseases	2445	5.2
		Candidiasis	207	0.4
		Leprosy	129	0.3
		Scabies	2915	6.1
		Tinea	5552	11.7
		Tinea versicolor	1500	3.2
		Viral diseases	2733	5.8

2	Dermatitis and eczema (L20–L30)	Dermatitis	7500	15.8
		Drug eruptions	257	0.5
		Nonspecific pruritis	623	1.3
		Seborrheic dermatitis	522	1.1

3	Papulosquamous disorders (L40–L45)	Lichen planus	533	1.1
		Psoriasis	1610	3.4

4	Urticaria and erythema (L50–L54)	Figurate erythemas	3	0.0
		Urticaria	1596	3.4

5	Radiation-related disorders of the skin and subcutaneous tissue (L55–L59)	Actinic keratosis	61	0.1

6	Disorders of skin appendages (L60–L75)	Acne	7933	16.7
		Alopecia	1363	2.9
		Palmoplantar hyperhidrosis	52	0.1
		Rosacea	64	0.1

7	Other disorders of the skin and subcutaneous tissue (L80–L99)	DLE	192	0.4
		Facial melanosis	312	0.7
		Hamartomas	2	0.0
		Ichthyosis	147	0.3
		IGH	102	0.2
		Keloid and hypertrophic scar	181	0.4
		Keratosis pilaris	65	0.1
		Melasma	1692	3.6
		P. Alba	766	1.6
		Palmoplantar keratoderma	419	0.9
		PIH	641	1.4
		Scleroderma	23	0.0
		Vasculitis	121	0.3
		Vitiligo	1582	3.3
		Nevus	239	0.5

8	Miscellaneous	Others	2881	6.1
		Not yet diagnosed	502	1.1

**Table 2:** Morbidity profile of dermatology out patients by age and gender in a tertiary care hospital in Srinagar, Garhwal, North India (2009–2014).

Morbidity as per ICD 10	Female *n* (%)			Male *n* (%)			Total *n* (%)
	<18 yrs	18–60 yrs	>60 yrs	<18 yrs	18–60 yrs	>60 yrs	
*Infections of the skin and subcutaneous tissue (L00–L08)*	*1200 (29.5)*	*4218 (23.7)*	*465 (38.8)*	*1906 (38.1)*	*7039 (40.1)*	*653 (35.5)*	*15481 (32.6)*
Fungal skin infections	374 (9.2)	2133 (12.0)	157 (13.1)	622 (12.4)	3686 (21.0)	287 (15.6)	7259 (15.3)
Scabies	232 (5.7)	699 (3.9)	85 (7.1)	471 (9.4)	1307 (7.4)	121 (6.6)	2915 (6.1)
Viral skin diseases	296 (7.3)	828 (4.7)	95 (7.9)	354 (7.1)	1017 (5.8)	143 (7.8)	2733 (5.8)
Bacterial skin diseases	298 (7.3)	558 (3.1)	128 (10.7)	459 (9.2)	1029 (5.9)	102 (5.5)	2574 (5.4)
*Dermatitis and eczema (L20–L30)*	*547 (13.5)*	*3636 (20.4)*	*420 (35.0)*	*663 (13.3)*	*2937 (16.7)*	*699 (38.0)*	*8902 (18.8)*
Dermatitis	510 (12.5)	3338 (18.8)	360 (30.0)	602 (12.0)	2644 (15.0)	568 (30.9)	8022 (16.9)
Nonspecific pruritis	26 (0.6)	203 (1.1)	52 (4.3)	45 (0.9)	185 (1.1)	112 (6.1)	623 (1.3)
*Papulosquamous disorders (L40–L45)*	*114 (2.8)*	*778 (4.4)*	*93 (7.8)*	*106 (2.1)*	*899 (5.1)*	*153 (8.3)*	*2143 (4.5)*
Psoriasis	93 (2.3)	533 (3.0)	59 (4.9)	85 (1.7)	735 (4.2)	105 (5.7)	1610 (3.4)
*Urticaria and erythema (L50–L54)*	*135 (3.3)*	*594 (3.3)*	*36 (3.0)*	*166 (3.3)*	*634 (3.6)*	*33 (1.8)*	*1599 (3.4)*
Urticaria	135 (3.3)	593 (3.3)	36 (3.0)	166 (3.3)	633 (3.6)	33 (1.8)	1596 (3.4)
*Radiation-related disorders of the skin and subcutaneous tissue (L55–L59)*	*0 (0.0)*	*25 (0.1)*	*7 (0.6)*	*2 (0.0)*	*17 (0.1)*	*10 (0.5)*	*61 (0.1)*
Actinic keratosis	0 (0.0)	25 (0.1)	7 (0.6)	2 (0.0)	17 (0.1)	10 (0.5)	61 (0.1)
*Disorders of skin appendages (L60–L75)*	*993 (24.4)*	*4179 (23.5)*	*13 (1.1)*	*964 (19.3)*	*3252 (18.5)*	*11 (0.6)*	*9412 (19.8)*
Acne	870 (21.4)	3624 (20.4)	6 (0.5)	882 (17.6)	2547 (14.5)	4 (0.2)	7933 (16.7)
Alopecia	102 (2.5)	510 (2.9)	6 (0.5)	69 (1.4)	670 (3.8)	6 (0.3)	1363 (2.9)
*Other disorders of the skin and subcutaneous tissue (L80–L99)*	*762 (18.8)*	*2985 (16.8)*	*93 (7.8)*	*812 (16.2)*	*1684 (9.6)*	*148 (8.0)*	*6484 (13.7)*
Melasma	18 (0.4)	1301 (7.3)	1 (0.1)	11 (0.2)	360 (2.0)	1 (0.1)	1692 (3.6)
Vitiligo	323 (7.9)	418 (2.3)	41 (3.4)	266 (5.3)	464 (2.6)	70 (3.8)	1582 (3.3)
P. alba	199 (4.9)	174 (1.0)	2 (0.2)	270 (5.4)	117 (0.7)	4 (0.2)	766 (1.6)
Postinflammatory hyperpigmentation	60 (1.5)	294 (1.7)	4 (0.3)	87 (1.7)	189 (1.1)	7 (0.4)	641 (1.4)
*Miscellaneous*	*313 (7.7)*	*1374 (7.7)*	*72 (6.0)*	*383 (7.7)*	*1107 (6.3)*	*134 (7.3)*	*3383 (7.1)*

*Total*	*4065*	*17789*	*1199*	*5002*	*17569*	*1841*	*47465*

**Table 3:** Morbidity profile of dermatology outpatients by residence district in a tertiary care hospital in Srinagar, Garhwal, North India (2009–2014).

Morbidity as per ICD 10	Rudraprayag	Pauri Garhwal	Chamoli	Tehri Garhwal	Others	Total
*Infections of the skin and subcutaneous tissue (L00–L08)*	*815 (35.2)*	*7807 (33.2)*	*338 (30.2)*	*882 (36.0)*	*5639 (31.2)*	*15481 (32.6)*
Fungal skin infections	384 (16.6)	3541 (15.1)	151 (13.5)	451 (18.4)	2732 (15.1)	7259 (15.3)
Scabies	152 (6.6)	1415 (6.0)	73 (6.5)	161 (6.6)	1114 (6.2)	2915 (6.1)
Viral skin diseases	143 (6.2)	1495 (6.4)	52 (4.6)	128 (5.2)	915 (3.1)	2733 (5.8)
Bacterial skin diseases	136 (5.9)	1356 (5.8)	62 (5.5)	142 (5.8)	878 (4.9)	2574 (5.4)
*Dermatitis and eczema (L20–L30)*	*423 (18.3)*	*4215 (17.9)*	*209 (18.7)*	*440 (17.9)*	*3615 (20.0)*	*8902 (18.8)*
Dermatitis	370 (16.0)	3702 (15.7)	191 (17.1)	390 (15.9)	3369 (18.7)	8022 (16.9)
Nonspecific pruritis	40 (1.7)	373 (1.6)	15 (1.3)	33 (1.3)	162 (0.9)	623 (1.3)
*Papulosquamous disorders (L40–L45)*	*146 (6.3)*	*924 (3.9)*	*86 (7.7)*	*107 (4.4)*	*880 (4.9)*	*2143 (4.5)*
Psoriasis	112 (4.8)	648 (2.8)	70 (6.3)	84 (3.4)	696 (3.9)	1610 (3.4)
*Urticaria and erythema (L50–L54)*	*77 (3.3)*	*928 (3.9)*	*39 (3.5)*	*80 (3.3)*	*475 (2.6)*	*1599 (3.4)*
Urticaria	76 (3.3)	927 (3.9)	38 (3.4)	80 (3.3)	475 (2.6)	1596 (3.4)
*Radiation-related disorders of the skin and subcutaneous tissue (L55–L59)*	*4 (0.2)*	*35 (0.1)*	*1 (0.1)*	*2 (0.1)*	*19 (0.1)*	*61 (0.1)*
Actinic keratosis	4 (0.2)	35 (0.1)	1 (0.1)	2 (0.1)	19 (0.1)	61 (0.1)
*Disorders of skin appendages (L60–L75)*	*359 (15.5)*	*4740 (20.1)*	*181 (16.2)*	*420 (17.1)*	*3712 (20.6)*	*9412 (19.8)*
Acne	284 (12.3)	3990 (17.0)	151 (13.5)	342 (13.9)	3166 (17.5)	7933 (16.7)
Alopecia	69 (3.0)	703 (3.0)	23 (2.1)	72 (2.9)	496 (2.7)	1363 (2.9)
*Other disorders of the skin and subcutaneous tissue (L80–L99)*	*330 (14.3)*	*2969 (12.6)*	*173 (15.5)*	*345 (14.1)*	*2667 (14.8)*	*6484 (13.7)*
Melasma	61 (2.6)	732 (3.1)	39 (3.5)	66 (2.7)	794 (4.4)	1692 (3.6)
Vitiligo	111 (4.8)	608 (2.6)	70 (6.3)	111 (4.5)	682 (3.8)	1582 (3.3)
P. alba	25 (1.1)	367 (1.6)	14 (1.3)	38 (1.5)	322 (1.8)	766 (1.6)
Postinflammatory hyperpigmentation	20 (0.9)	380 (1.6)	10 (0.9)	46 (1.9)	185 (1.0)	641 (1.4)
*Miscellaneous*	*160 (6.9)*	*1909 (8.1)*	*92 (8.2)*	*177 (7.2)*	*1045 (5.8)*	*3383 (7.1)*

*Total*	*2314 (4.8)*	*23527 (49.6)*	*1119 (2.4)*	*2453 (5.2)*	*18052 (38.0)*	*47465*

## Abbreviations

OPD: Outpatient department.
